# Genome sequence and description of *Bacteroides timonensis* sp. nov.

**DOI:** 10.4056/sigs.5389564

**Published:** 2014-03-15

**Authors:** Dhamodharan Ramasamy, Jean-Christophe Lagier, Morgane Rossi-Tamisier, Anne Pfleiderer, Caroline Michelle, Carine Couderc, Didier Raoult, Pierre-Edouard Fournier

**Affiliations:** 1 Unité de Recherche sur les Maladies Infectieuses et Tropicales Emergentes, Institut Hospitalo-Universitaire Méditerranée-Infection, Faculté de médecine, Aix-Marseille Université, Marseille, France; 2 King Fahd Medical Research Center, King Abdul Aziz University, Jeddah, Saudi Arabia

**Keywords:** *Bacteroides timonensis*, genome, culturomics, taxono-genomics

## Abstract

*Bacteroides timonensis* strain AP1^T^ (= CSUR P194 = DSM 26083) is the type strain of *B. timonensis* sp. nov. This strain, whose genome is described here, was isolated from the fecal flora of a 21-year-old French Caucasoid female who suffered from severe anorexia nervosa. *Bacteroides timonensis* is a Gram-negative, obligate anaerobic bacillus. Here we describe the features of this organism, together with the complete genome sequence and annotation. The 7,130,768 bp long genome (1 chromosome, no plasmid) exhibits a G+C content of 43.3% and contains 5,786 protein-coding and 59 RNA genes, including 2 rRNA genes.

## Introduction

*Bacteroides timonensis* strain AP1^T^ (= CSUR P194 = DSM 26083) is the type strain of *B. timonensis* sp. nov. This bacterium was isolated from the stool sample of a 21-year-old French Caucasoid female in an effort of cultivating individually all bacterial species within human feces [1]. It is a Gram-negative, anaerobic, indole-positive rod-shaped bacillus.

The conventional genetic parameters used in the delineation of bacterial species include 16S rRNA sequence identity and phylogeny [2,3], genomic G + C content diversity and DNA–DNA hybridization (DDH) [4,5]. These tools have limitations, notably because their cutoff values vary across species or genera [6]. With the introduction of high-throughput sequencing techniques [7], a wealth of genomic data was made available for many bacterial species. We recently proposed to include genomic data in a polyphasic approach to describe new bacterial taxa (taxono-genomics) [8]. This strategy combines phenotypic characteristics, notably the MALDI-TOF MS spectrum, and genomic analysis [8-37].

Here, we present a summary classification and a set of features for *B. timonensis* sp. nov. strain AP1^T^ (= CSUR P194 = DSM 26083) together with the description of the complete genome sequencing and annotation. These characteristics support the circumscription of the type species, *B. timonensis*.

The genus *Bacteroides* (Castellani and Chalmers 1919) was created in 1919 [38]. Currently, it is one of the largest genera among the human gut microbiota [39], and consists of 91 species and 5 subspecies with validly published names [40]. *Bacteroides* species are Gram-negative, non-spore-forming, non-motile and anaerobic rods that are generally isolated from the gastrointestinal tract of mammals [41]. They have symbiotic relationships with humans and play many beneficial roles on normal intestinal physiology and function. Several *Bacteroides* species are identified as opportunistic pathogens when isolated from anaerobic infections [42].

## Classification and features

A stool sample was collected from 21-year-old French Caucasoid female who suffered from severe restrictive anorexia nervosa from the age of 12 years. At the time of sample collection, she had been hospitalized for recent aggravation of her medical condition (BMI: 10.4 kg/m^2^). The patient’s written consent and the agreement of the local ethics committee of the IFR48 (Marseille, France) were obtained under agreement number 09-022. The feces sample of this patient was stored at -80°C immediately after collection. Strain AP1^T^ (Table 1) was isolated in November 2011 after 1 month of incubation in Columbia agar (BioMerieux, Marcy l’Etoile, France). Several other new bacterial species were isolated from this stool specimen using various culture conditions.

**Table 1:** Classification and general features of *Bacteroides timonensis* strain AP1^T^ according to the MIGS recommendations [43]

**MIGS ID**	**Property**	**Term**	**Evidence code^a^**
	Current classification	Domain *Bacteria*	TAS [44]
		Phylum *Bacteroidetes*	TAS [45,46]
		Class *Bacteroidia*	TAS [45,47]
		Order *Bacteroidales*	TAS [45,48]
		Family *Bacteroidaceae*	TAS [49,50]
		Genus *Bacteroides*	IDA [49,51-54]
		Species *Bacteroides timonensis*	IDA
		Type strain AP1^T^	IDA
	Gram stain	Negative	IDA
	Cell shape	Rod	IDA
	Motility	Non motile	IDA
	Sporulation	Non sporulating	IDA
	Temperature range	Mesophile	IDA
	Optimum temperature	37°C	IDA
MIGS-6.3	Salinity	Unknown	IDA
MIGS-22	Oxygen requirement	Anaerobic	IDA
	Carbon source	Unknown	IDA
	Energy source	Unknown	IDA
MIGS-6	Habitat	Human gut	IDA
MIGS-15	Biotic relationship	Free living	IDA
MIGS-14	Pathogenicity Biosafety level Isolation	Unknown 2 Human feces	
MIGS-4	Geographic location	France	IDA
MIGS-5	Sample collection time	November 2011	IDA
MIGS-4.1	Latitude	43.296482	IDA
MIGS-4.1	Longitude	5.36978	IDA
MIGS-4.3	Depth	surface	IDA
MIGS-4.4	Altitude	0 m above sea level	IDA

Evidence codes - IDA: Inferred from Direct Assay; TAS: Traceable Author Statement (i.e., a direct report exists in the literature); NAS: Non-traceable Author Statement (i.e., not directly observed for the living, isolated sample, but based on a generally accepted property for the species, or anecdotal evidence). These evidence codes are from the Gene Ontology project [55]. If the evidence is IDA, then the property was directly observed for a live isolate by one of the authors or an expert mentioned in the acknowledgements.

**Figure 1:**
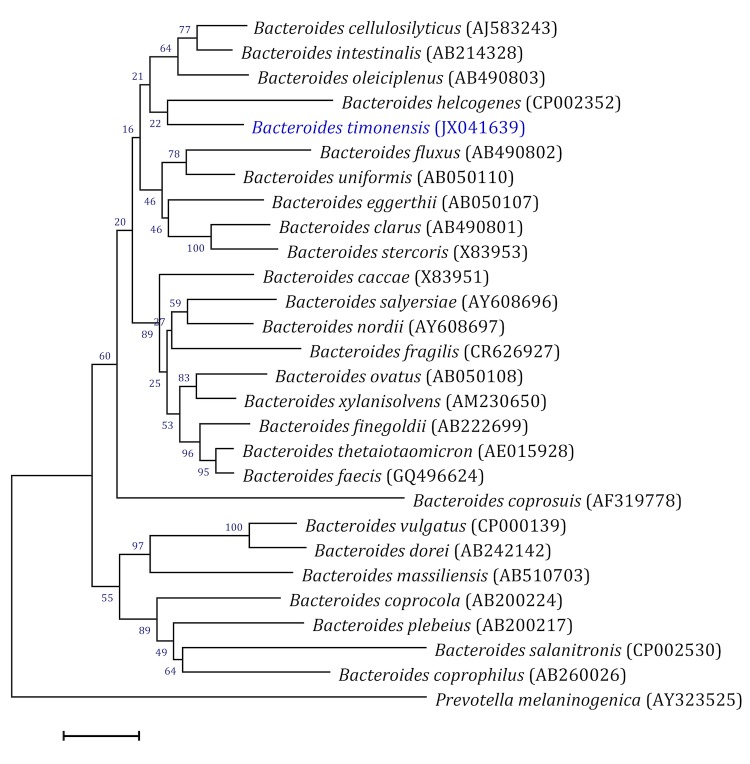
Phylogenetic tree highlighting the position of *Bacteroides timonensis* strain AP1^T^ relative to other type strains within the *Bacteroides* genus. GenBank accession numbers are indicated in parentheses. Sequences were aligned using CLUSTALW, and phylogenetic inferences were obtained using the maximum-likelihood method within the MEGA software. Numbers at the nodes are percentages of bootstrap values obtained from 500 replicates. *Prevotella melaninogenica* was used as outgroup. The scale bar represents a 2% nucleotide sequence divergence.

When compared to sequences available in GenBank, the 16S rRNA gene sequence of *B. timonensis* strain AP1^T^ (GenBank accession number JX041639) exhibited an identity of 97.00% with *Bacteroides cellulosilyticus* (Figure 1). This value was the highest similarity observed, but was lower than the 97.8% 16S rRNA gene sequence threshold recommended by Stackebrandt and Ebers (2006) to delineate a new species without carrying out DNA-DNA hybridization [3], and was in the 74. 8 to 98.7% range of 16S rRNA identity values observed among 41 *Bacteroides* species with validly published names [56].

**Figure 2:**
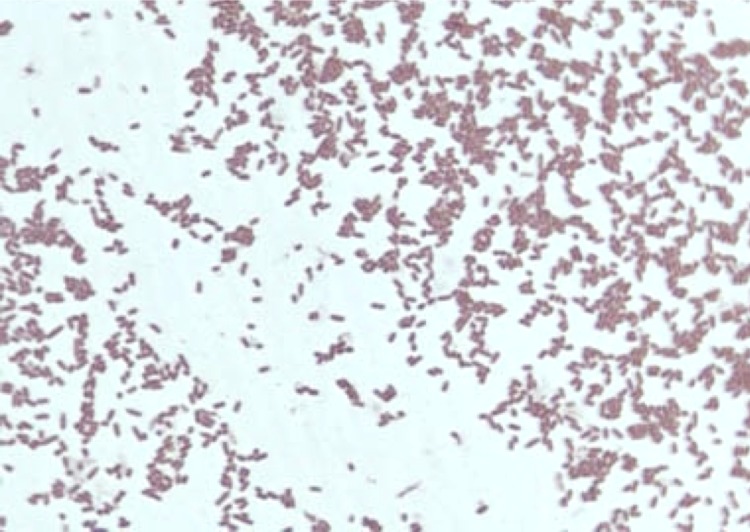
Gram staining of *B. timonensis* strain AP1^T^

**Figure 3:**
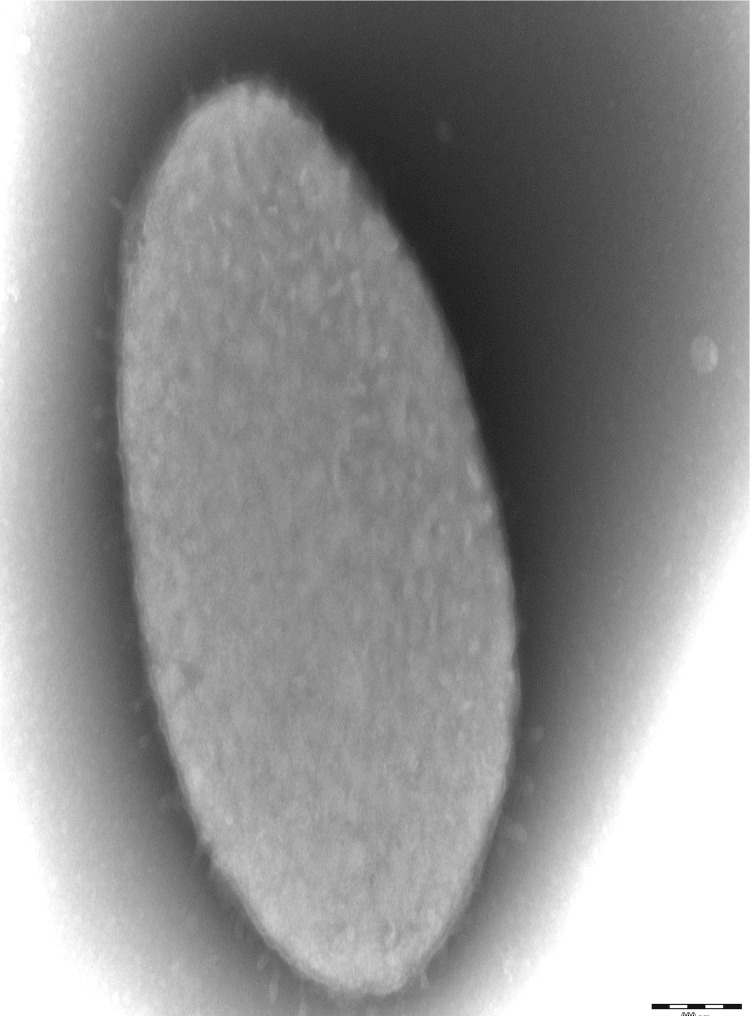
Transmission electron microscopy of *B. timonensis* strain AP1^T^, made using a Morgani 268D (Philips) at an operating voltage of 60kV.The scale bar represents 200 nm

Four different growth temperatures (25, 30, 37, 45°C) were tested; growth occurred between 25 and 37°C, but optimal growth was observed at 37°C, 24 hours after inoculation. No growth occurred at 45°C. Colonies were translucent and approximately 0.3 mm in diameter on 5% sheep blood-enriched Columbia agar (BioMerieux). Growth of the strain was tested in the same agar under anaerobic and microaerophilic conditions using GENbag anaer and GENbag microaer systems, respectively (BioMerieux), and under aerobic conditions, with or without 5% CO_2_. Growth was observed under anaerobic and microaerophilic conditions, and only weakly with 5% CO_2_. No growth occurred under aerobic condition without CO_2_. Gram staining showed short Gram-negative rods unable to form spores (Figure 1). A motility test was negative. Cells grown on agar are translucent and exhibit a mean diameter of 0.88 µm in electron microscopy (Figure 2, Figure 3).

Strain AP1^T^ exhibited catalase but no oxidase activity (Table 2). Using an API Rapid ID 32A strip (BioMerieux), positive reactions were obtained for arginine dihydrolase, α-galactosidase, β-galactosidase, α-glucosidase, β-glucosidase, α-arabinosidase, N-acetyl-β-glucosaminidase, glutamic acid decarboxylase, α-fucosidase, nitrate reduction, indole production, alkaline phosphatase, proline arylamidase, leucyl glycine arylamidase, alanine arylamidase, glutamyl glutamic acid arylamidase, and fermentation of mannose and raffinose. Weak activities were observed for glycine arylamidase and serine arylamidase. Negative reactions were obtained for urease, β-galctosidase-6-phosphatase, β-glucuronidase, arginine arylamidase, phenylalanine arylamidase, leucine arylamidase, pyroglutamic acid arylamidase, tyrosine arylamidase and histidine arylamidase. Using an API 50CH strip (Biomerieux), strain AP1^T^ was asaccharolytic. *B. timonensis* is susceptible to amoxicillin-clavulanate, ceftriaxon, imipenem, trimethoprim-sulfamethoxazole, metronidazole and doxycycline but resistant to amoxicillin, vancomycin and gentamicin. By comparison with other *Bacteroides* species, *B. timonensis* differed in production of indole, nitrate reductase, β-galactosidase and acidification of sugars.

**Table 2:** Differential characteristics of *Bacteroides* species [57-67]^†^.

**Properties**	*B. timonensis*	*B. cellulosyticus*	*B. intestinalis*	*B. fragilis*	*B. vulgatus*	*B. thetaiotaomicron*	*B. salanitronis*	*B. helcogenes*	*B. finegoldii*	*B. uniformis*
Cell diameter (µm)	0.88	2-5	1-3	1.3	0.5-0.8	0.7-2	2-3	1-2	1-2	0.5-2
Oxygen requirement	anaerobic	anaerobic	anaerobic	anaerobic	anaerobic	anaerobic	anaerobic	anaerobic	anaerobic	anaerobic
Gram stain	–	–	–	–	+	–	–	–	–	–
Salt requirement	+	+	+	+	na	na	na	na	+	+
Motility	–	–	–	–	–	–	–	–	–	–
Endospore formation	–	–	–	+	na	–	–	–	–	–
Indole	+	+	+	–	–	+	–	–	–	+
**Production of**										
Alkaline phosphatase	+	+	+	Na	+	+	+	na	+	na
Catalase	+	–	+	+	na	+	–	–	na	–
Oxidase	–	+	na	+	na	na	na	na	na	na
Nitrate reductase	+	na	–	na	–	+	–	–	–	–
Urease	–	na	–	na	na	–	na	–	–	na
β-galactosidase	+	–	+	na	–	+	+	+	+	+
N-acetyl-glucosamine	+	+	+	na	na	+	na	na	+	+
**Acid from**										
L-Arabinose	–	w	+	–	–	+	+	–	–	+
Ribose	–	+	na	–	+	na	na	–	na	na
Mannose	–	+	+	+	+	+	+	+	+	+
Mannitol	–	–	–	–	–	–	–	–	–	–
Sucrose	–	+	+	+	+	+	+	+	+	+
D-glucose	–	+	+	+	+	+	+	+	+	+
D-fructose	–	+	+	+	+	+		+	na	na
D-maltose	–	+	+	+	+	+	+	+	+	+
D-lactose	–	w	+	+	+	+	+	+	+	+
**Habitat**	human gut	human gut	human gut	human gut	human gut	human gut	human gut	pig gut	human gut	human gut

^†^*Bacteroides timonensis* strain AP1^T^, *B. cellulosilyticus* strain DSM 14838, *B. intestinalis* strain DSM 17393, *B. fragilis* strain YCH46, *B. vulgatus* strain ATCC 8482, *B. thetaiotaomicron* strain VPI-5482, *B. salanitronis* strain DSM 18170, *B. helcogenes* strain P 36-108, *B. finegoldii* strain DSM 17565 and *B. uniformis* strain ATCC 8492

na = data not available; w = weak, v = variable reaction

**Figure 4:**
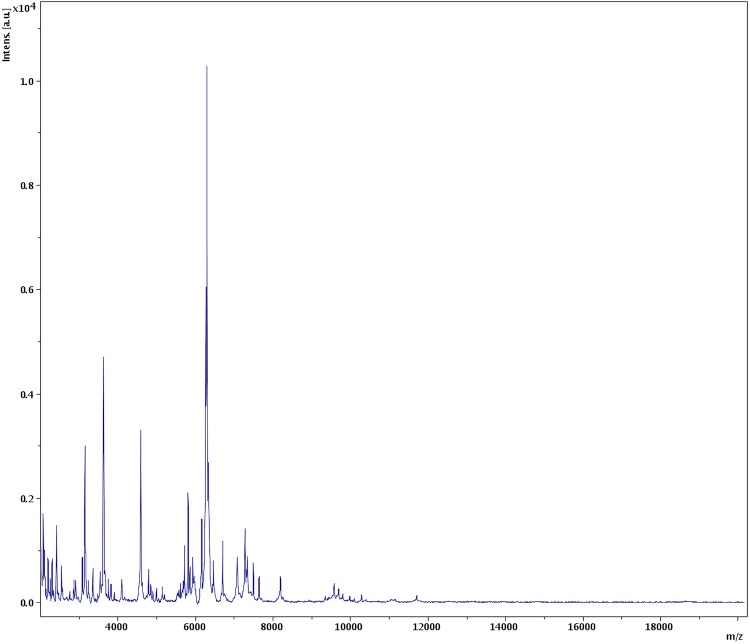
Reference mass spectrum from *B. timonensis* strain AP1^T^. Spectra from 12 individual colonies were compared and a reference spectrum was generated.

**Figure 5:**
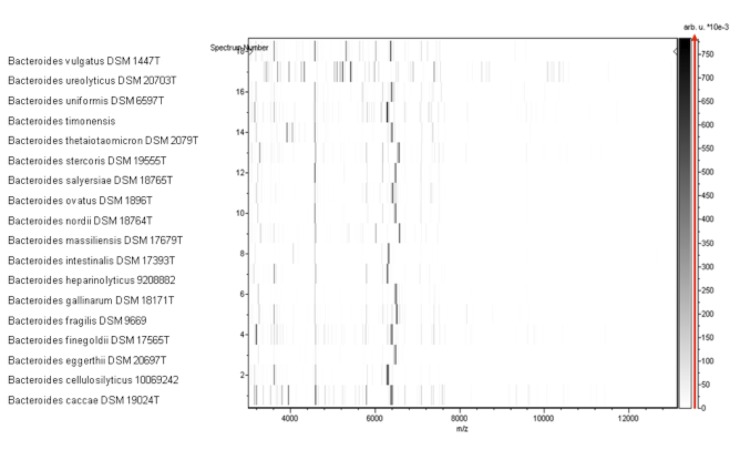
Gel view comparing *B. timonensis* strain AP1^T^ to other *Bacteroides* species. The gel view displays the raw spectra of loaded spectrum files as a pseudo-electrophoretic gel. The x-axis records the m/z value. The left y-axis displays the running spectrum number originating from subsequent spectra loading. The peak intensity is expressed by a grey scale scheme code. The grey scale bar on the right y-axis indicate the relation between the shade of grey a peak is displayed with and the peak intensity in arbitrary units. Displayed species are detailed in the left column.

Matrix-assisted laser-desorption/ionization time-of-flight (MALDI-TOF) MS protein analysis was carried out as previously described [68]. Briefly, a pipette tip was used to pick one isolated bacterial colony from a culture agar plate and spread it as a thin film on a MTP 384 MALDI-TOF target plate (Bruker Daltonics, Leipzig, Germany). Twelve distinct deposits from twelve isolated colonies were performed for strain AP1^T^. Each smear was overlaid with 2 µL of matrix solution (saturated solution of alpha-cyano-4-hydroxycinnamic acid) in 50% acetonitrile, 2.5% tri-fluoracetic acid, and allowed to dry for 5 minutes. Measurements were performed with a Microflex spectrometer (Bruker). Spectra were recorded in the positive linear mode for the mass range of 2,000 to 20,000 Da (parameter settings: ion source 1 (ISI), 20kV; IS2, 18.5 kV; lens, 7 kV). A spectrum was obtained after 675 shots with variable laser power. The time of acquisition was between 30 seconds and 1 minute per spot. The twelve AP1^T^ spectra were imported into the MALDI BioTyper software (version 2.0, Bruker) and analyzed by standard pattern matching (with default parameter settings) against the main spectra of 3,769 bacteria, including 129 spectra from 98 *Bacteroides* species. The method of identification included the m/z from 3,000 to 15,000 Da. For every spectrum, a maximum of 100 peaks were compared with spectra in database. The resulting score enabled the identification of tested species, or not: a score ≥ 2 with a validly published species enabled identification at the species level, a score ≥ 1.7 but < 2 enabled identification at the genus level, and a score < 1.7 did not enable any identification. No significant MALDI-TOF score was obtained for strain AP1^T^ against the Bruker database, suggesting that our isolate was not a member of a known species. We added the spectrum from strain AP1^T^ to our database (Figure 4). Finally, the gel view showed the spectral differences with other members of the genus *Bacteroides* (Figure 5).

## Genome sequencing information

### Genome project history

The organism was selected for sequencing on the basis of its phylogenetic position and 16S rRNA gene sequence similarity to members of the genus *Bacteroides*, and is part of a study of the human digestive flora aiming at isolating all bacterial species within human feces [1]. It was the ninety-ninth genome of a *Bacteroides* species and the first genome of *B. timonensis* sp. nov. The GenBank accession number is CBVI000000000 and consists of 211 contigs. Table 3 shows the project information and its association with MIGS version 2.0 compliance [43].

**Table 3:** Project information

**MIGS ID**	**Property**	**Term**
MIGS-31	Finishing quality	High-quality draft
MIGS-28	Libraries used	454 GS paired-end 3-kb library
MIGS-29	Sequencing platform	454 GS FLX Titanium
MIGS-31.2	Fold coverage	35.76
MIGS-30	Assemblers	gsAssembler
MIGS-32	Gene calling method	PRODIGAL

### Growth conditions and DNA isolation

*B. timonensis* sp. nov., strain AP1^T^ (= CSUR P194 = DSM 26083) was grown on 5% sheep blood-enriched Columbia agar (BioMerieux) at 37°C in anaerobic atmosphere. Bacteria grown on four Petri dishes were harvested and resuspended in 4x100µL of TE buffer. Then, 200µL of this suspension was diluted in 1ml TE buffer for lysis treatment that included a 30- minute incubation with 2.5 µg/µL lysozyme at 37°C, followed by an overnight incubation with 20 µg/µL proteinase K at 37°C. Extracted DNA was then purified using 3 successive phenol-chloroform extractions and ethanol precipitation at -20°C overnight. After centrifugation, the DNA was resuspended in 160 µL TE buffer. The yield and concentration was measured by the Quant-it Picogreen kit (Invitrogen) on the Genios-Tecan fluorometer at 88.6 ng/µl.

### Genome sequencing and assembly

Five µg of DNA was mechanically fragmented on Covaris device (KBioScience-LGC Genomics, Teddington, UK) using miniTUBE-blue. The DNA fragmentation was visualized through an Agilent 2100 BioAnalyzer on a DNA labchip 7500 with an average size of 2.950kb. A 3 kb paired-end library was constructed according to the 454 GS FLX Titanium paired-end protocol (Roche). Circularization and nebulization were performed and generated a pattern with a mean size of 513 bp. After PCR amplification through 17 cycles followed by double size selection, the single stranded paired-end library was quantified with the Quant-it Ribogreen kit (Invitrogen) on the Genios Tecan fluorometer at 243 pg/µL. The library concentration equivalence was calculated as 8.69 x 10^8^ molecules/µL. The library was stored at -20°C until further use.

The paired-end library was clonally amplified with 0.5cpb and 1cbp in 8 SV-emPCR reactions with the GS Titanium SV emPCR Kit (Lib-L) v2 (Roche). The yields of the emPCR reactions were 4.65 and 7.29% respectively, within the recommended range of 5 to 20% from the Roche procedure. Approximately 790,000 beads were loaded on a 1/4 region of a GS Titanium PicoTiterPlate PTP Kit 70×75 and sequenced with the GS Titanium Sequencing Kit XLR70 (Roche). The run was performed overnight and then analyzed on the cluster through the gsRunBrowser and Newbler assembler (Roche). A total of 802,249 passed filter wells were obtained and generated 255Mb with a length average of 314 bp. These sequences were assembled using Newbler (Roche) with 90% identity and 40bp as overlap. The final assembly identified 63 scaffolds and 211 large contigs (>1,500bp) generating a genome size of 7.13 Mb which corresponds to a coverage of 35.76× genome equivalent.

### Genome annotation

Open Reading Frames (ORFs) were predicted using Prodigal [69] with default parameters. However, the predicted ORFs were excluded if they spanned a sequencing gap region. The predicted bacterial protein sequences were searched against the GenBank [70] and Clusters of Orthologous Groups (COG) databases using BLASTP. The tRNAs and rRNAs were predicted using the tRNAScan-SE [71] and RNAmmer [72] tools, respectively. Signal peptides and numbers of transmembrane helices were predicted using SignalP [73] and TMHMM [74], respectively. Mobile genetic elements were predicted using PHAST [75] and RAST [76]. ORFans were identified if their BLASTP *E*-value was lower than 1e-03 for alignment length greater than 80 amino acids. If alignment lengths were smaller than 80 amino acids, we used an *E*-value of 1e-05. Such parameter thresholds have already been used in previous works to define ORFans. Artemis [77] and DNA Plotter [78] were used for data management and visualization of genomic features, respectively. The Mauve alignment tool (version 2.3.1) was used for multiple genomic sequence alignment [79].

 To estimate the mean level of nucleotide sequence similarity at the genome level between *B. timonensis* and 9 other members of the genus *Bacteroides* (Table 6), we used the Average Genomic Identity Of gene Sequences (AGIOS) in-house software [8]. Briefly, this software uses the Proteinortho software [80] for the pairwise detection of orthologous proteins between genomes, then retrieves the corresponding genes and determines the mean percentage of nucleotide sequence identity among orthologous ORFs using the Needleman-Wunsch global alignment algorithm. *B. timonensis* strain AP1^T^ was compared to *B. intestinalis* strain DSM 17393 (GenBank accession number NZ_ABJL00000000), *B. cellulosilyticus* strain DSM 14838 (NZ_ACCH00000000), *B. fragilis* strain YCH46 (NC_006347), *B. vulgatus* strain ATCC 8482 (NC_009614), *B. thetaiotaomicron* strain VPI-5482 (NC_004663), *B. salanitronis* strain DSM 18170 (NC_015164), *B. helcogenes* strain P36-108 (NC_014933), *B. finegoldii* strain DSM 17565 (NZ_ABXI00000000) and *B. uniformis* strain ATCC 8492 (AAYH00000000).

## Genome properties

**Figure 6:**
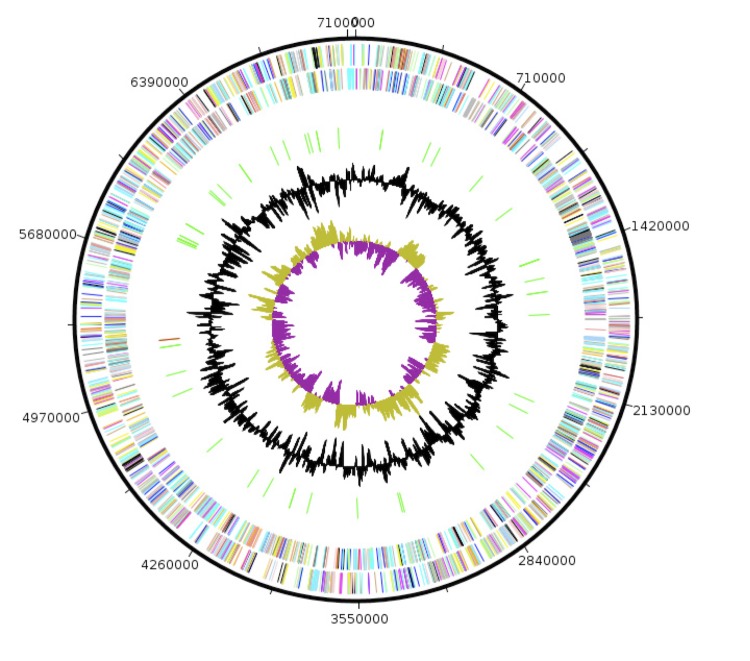
Graphical circular map of the chromosome. From the outside in: open reading frames oriented in the forward (colored by COG categories) direction, open reading frames oriented in the reverse (colored by COG categories) direction, RNA operon (red), and tRNAs (green), GC content plot, and GC skew (purple: negative values, olive: positive values)..

The genome is 7,130,768 bp long (1 chromosome, but no plasmid) with a 43.3% G+C content (Figure 6 and Table 4). Of the 5,845 predicted genes, 5,786 were protein-coding genes and 59 were RNAs, including 1 complete rRNA operon. A total of 3,111 genes (53.22%) were assigned a putative function and 3,283 genes were identified as ORFans (56.16%). Strain AP1^T^ possesses a variety of mobile genetic elements. These include 6 prophages of 13.70, 14.60, 10.51, 8.18, 9.91 and 12.79 Kb, respectively) and 91 transposable elements belonging to 18 transposon families that include the putative mobilization protein BF0133, the putative conjugative transposon mobilization protein BF0132, the hypothetical protein clustered with conjugative transposons BF0131, TraA-CTn, TraB-CTn,TraD-CTn,TraE-CTn,TraF-CTn,TraG-CTn,TraH-CTn,TraI-CTn,TraJ-CTn,TraK-CTn,TraL-CTn,TraM-CTn,TraN-CTn,TraO-CTn and TraQ-CTn. The properties and statistics of the genome are summarized in Tables 4 and 5. The distribution of genes into COGs functional categories is presented in Table 5.

**Table 4:** Nucleotide content and gene count levels of the genome

**Attribute**	**Value**	% of total^a^
Genome size (bp)	7,130,768	
DNA coding region (bp)	6,434,142	90.23
DNA G+C content (bp)	3,087,622	43.30
Number of replicons	1	
Extra chromosomal element	0	
Total genes	5,845	100
RNA genes	59	1.01
Protein-coding genes	5,786	98.99
Genes with function prediction	3,111	53.22
Genes assigned to COGs	2,820	48.24
Genes with peptide signals	435	7.44
Genes with transmembrane helices	456	7.80

^a^ The total is based on either the size of the genome in base pairs or the total number of protein-coding genes in the annotated genome

**Table 5:** Number of genes associated with the 25 general COG functional categories

**Code**	**Value**	**%age**^a^	**Description**
J	156	2.66	Translation
A	0	0	RNA processing and modification
K	234	4.00	Transcription
L	200	3.42	Replication, recombination and repair
B	0	0	Chromatin structure and dynamics
D	27	0.46	Cell cycle control, mitosis and meiosis
Y	0	0	Nuclear structure
V	107	1.83	Defense mechanisms
T	240	4.22	Signal transduction mechanisms
M	361	6.17	Cell wall/membrane biogenesis
N	5	0.08	Cell motility
Z	0	0	Cytoskeleton
W	0	0	Extracellular structures
U	65	1.11	Intracellular trafficking and secretion
O	89	1.52	Posttranslational modification, protein turnover, chaperones
C	168	2.87	Energy production and conversion
G	369	6.31	Carbohydrate transport and metabolism
E	212	3.62	Amino acid transport and metabolism
F	73	1.25	Nucleotide transport and metabolism
H	130	2.22	Coenzyme transport and metabolism
I	87	1.48	Lipid transport and metabolism
P	202	3.42	Inorganic ion transport and metabolism
Q	47	0.80	Secondary metabolites biosynthesis, transport and catabolism
R	518	8.86	General function prediction only
S	197	3.37	Function unknown
-	2966	51.26	Not in COGs

^a^ The total is based on the total number of protein-coding genes in the annotated genome

## Genome comparison with other *Bacteroides* genomes

**Figure 7:**
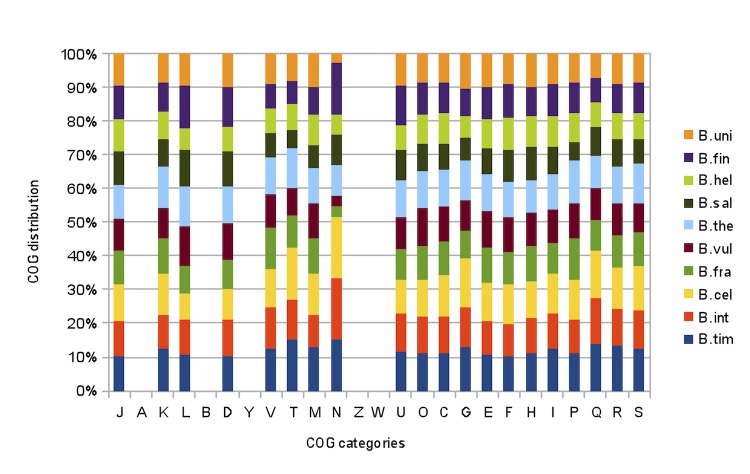
Distribution of predicted genes of *B. timonensis* and 9 other *Bacteroides* species into COG categories. B. uni = *B. uniformis*, B. fin = *B. finegoldii*, B. hel = *B. helcogenes*, B. sal = *B. salanitronis*, B. the = *B. thetaiotaomicron*, B. vul = *B. vulgatus*, B. fra = *B. fragilis*, B. cel = *B. cellulosilyticus*, B. int = *B. intestinalis*, B. tim *= B. timonensis*.

Here, we compare the genome of *B. timonensis* with those of *B. intestinalis*, DSM 17393, *B. cellulosilyticus* DSM 14838, *B. fragilis* YCH46, *B. vulgatus* ATCC 8482, *B. thetaiotaomicron* VPI-5482, *B. salanitronis* DSM 18170, *B. helcogenes* P 36-108, *B. finegoldii* DSM 17565 and *B. uniformis* ATCC 8492. The draft genome of *B. timonensis* (7.13Mb) is larger than all other studied genomes (Table 6A). It also exhibits a higher G+C content than all other genomes except *B. salanitronis*, *B. helcogenes* and *B. uniformis* (43.3, 46.4, 44.7 and 46.4%, respectively). *B. timonensis* has a higher gene content (5,786) than any other compared genome. The distribution of genes into COG categories was similar in all 10 compared genomes except in the N category (cell motility) for which *B. fragilis*, *B. vulgatus, B. salanitronis, B. helcogenes* and *B. uniformis* were underrepresented (Figure 7). In addition, *B. timonensis* shared 2,956, 3,081, 2,159, 2,099, 2,379, 1,721, 2,001, 2,039 and 2,268 orthologous genes with *B. intestinalis, B. cellulosilyticus*, *B. fragilis*, *B. vulgatus, B. thetaiotaomicron*, *B. salanitronis, B. helcogenes*, *B. finegoldii* and *B. uniformis*, respectively. Among compared genomes except *B. timonensis*, AGIOS values ranged from 70.16 between *B. salitronis* and *B. cellulosilyticus* to 88.16% between *B. intestinalis* and *B. cellulosilyticus*. When *B. timonensis* was compared to other species, AGIOS values ranged from 70.29 with *B. salitronis* to 93.61% with *B. cellulosilyticus* (Table 6B).

**Table 6A:** Genomic comparison of *B. timonensis* with 9 other *Bacteroides* species^†^.

**Species**	**Strain**	**Genome accession** **number**	**Genome size (Mb)**	**G+C content**
*B. timonensis*	AP1	CBVI010000000	7.13	43.3
*B. intestinalis*	DSM 17393	NZ_ABJL00000000	6.05	42.8
*B. cellulosilyticus*	DSM 14838	NZ_ACCH00000000	6.87	42.7
*B. fragilis*	YCH46	NC_006347	5.28	43.2
*B. vulgatus*	ATCC 8482	NC_009614	5.16	42.2
*B. thetaiotaomicron*	VPI-5482	NC_004663	6.26	42.8
*B. salanitronis*	DSM 18170	NC_015164	4.24	46.4
*B. helcogenes*	P 36-108	NC_014933	4.0	44.7
*B. finegoldii*	DSM 17565	NZ_ABXI00000000	4.89	42.9
*B. uniformis*	ATCC 8492	AAYH00000000	4.72	46.4

**^†^**Species, Strain, GenBank accession number, genome size and G+C content of all compared genomes.

**Table 6B:** Genomic comparison of *B. timonensis* with 9 other *Bacteroides* species^†^.

	***B. tim***	***B.int***	***B. cel***	***B. fra***	***B. vul***	***B. the***	***B. sal***	***B. hel***	***B. fin***	***B. uni***
***B. tim***	**5,786**	2,956	3,081	2,159	2,099	2,379	1,721	2,001	2,039	2,268
***B. int***	87.73	**4,911**	2,967	2,085	2,036	2,361	1,667	1,963	2,066	2,278
***B. cell***	93.61	88.16	**5,719**	2,130	2,078	2,380	1,655	1,990	2,017	2,231
***B. fra***	73.76	74.43	73.92	**4,184**	1,927	2,174	1,517	1,893	1,880	1,995
***B. vul***	71.91	71.74	71.48	71.87	**4,066**	2,100	1,638	1,743	1,859	1,898
***B. the***	73.99	74.65	73.87	75.42	72.21	**4,778**	1,601	1,891	2,191	2,039
***B. sal***	70.29	70.65	70.16	70.35	72.18	70.50	**3,553**	1,466	1,580	1,584
***B. hel***	76.40	76.51	76.41	74.15	71.62	73.64	70.68	**3,244**	1,703	1,930
***B. fin***	74.28	75.01	74.45	75.72	72.22	81.24	70.77	73.99	**4,485**	1,920
***B. uni***	77.08	76.83	76.80	74.25	72.45	74.36	71.32	79.37	74.77	**4,663**

† numbers of orthologous proteins shared between genomes (above diagonal), AGIOS values (below diagonal) and numbers of proteins per genome (bold numbers).

*B. tim = B. timonensis*, *B. int* = *B. intestinalis*, *B. cel* = *B. cellulosilyticus*, *B. fra* = *B. fragilis*, *B. vul* = *B. vulgatus*, *B. the* = *B. thetaiotaomicron*, *B. sal* = *B. salanitronis*, *B. hel* = *B. helcogenes*, *B. uni* = *B. uniformis*, *B. fin* = *B. finegoldii.*

## Conclusion

On the basis of phenotypic, phylogenetic and genomic analyses (taxono-genomics), we formally propose the creation of *Bacteroides timonensis* sp. nov. that contains strain AP1^T^. This strain was isolated from the fecal flora of a 21-year-old woman who suffered from severe anorexia nervosa.

## Description of *B. timonensis* sp. nov.

*Bacteroides timonensis* (tim.o.nen’sis. L. masc. adj. timonensis, of Timone, the name of the hospital where strain AP1^T^ was first cultivated).

Colonies are translucent and 0.3 mm in diameter on blood-enriched Columbia agar. Cells are rod-shaped with a mean diameter of 0.88 µm. Optimal growth is achieved anaerobically, although the strain is able to grow under microaerophilic conditions, and weakly with 5% CO_2_. Growth occurs between 25°C and 37°C, with optimal growth at 37°C. Cells stain Gram-negative and are not motile. Positive reactions for catalase, arginine dihydrolase, α-galactosidase, β-galactosidase, α-glucosidase, β-glucosidase, α-arabinosidase, N-acetyl-β-glucosaminidase, glutamic acid decarboxylase, α-fucosidase, nitrate reduction, indole production, alkaline phosphatase, proline arylamidase, leucyl glycine arylamidase, alanine arylamidase, glutamyl glutamic acid arylamidase, and fermentation of mannose and raffinose.

Weak activities are observed for glycine arylamidase and serine arylamidase. Negative reactions are obtained for urease, β-galctosidase-6-phosphatase, β-glucuronidase, arginine arylamidase, phenylalanine arylamidase, leucine arylamidase, pyroglutamic acid arylamidase, tyrosine arylamidase and histidine arylamidase. Using an API 50CH strip (Biomerieux), strain AP1^T^ is asaccharolytic. Cells are susceptible to susceptible to amoxicillin-clavulanate, ceftriaxone, imipenem, trimethoprim-sulfamethoxazole, metronidazole and doxycycline but resistant to amoxicillin, vancomycin and gentamicin.

The 16S rRNA and genome sequences are deposited in GenBank under accession numbers JX041639 and CBVI000000000, respectively. The G+C content of the genome is 43.3%. The habitat of the organism is the digestive tract. The type strain AP1^T^ (= CSUR P194 = DSMZ 26083) was isolated from the fecal flora of a French Caucasoid female who suffered from a severe restrictive form of anorexia nervosa. This strain has been found in Marseille, France.
